# Impact Assessment of Cadmium Toxicity and Its Bioavailability in Human Cell Lines (Caco-2 and HL-7702)

**DOI:** 10.1155/2014/839538

**Published:** 2014-02-16

**Authors:** Rukhsanda Aziz, M. T. Rafiq, Jie Yang, Di Liu, Lingli Lu, Zhenli He, M. K. Daud, Tingqiang Li, Xiaoe Yang

**Affiliations:** ^1^Ministry of Education Key, Laboratory of Environmental Remediation and Ecological Health, College of Environmental and Resource Sciences, Zhejiang University, Hangzhou 310058, China; ^2^Department of Environmental Sciences, International Islamic University, Islamabad 44000, Pakistan; ^3^College of Architecture and Environment, Sichuan University, Chengdu 610065, China; ^4^Indian River Research and Education Center, Institute of Food and Agricultural Sciences, University of Florida, Fort Pierce, FL 34945, USA; ^5^Department of Biotechnology & Genetic Engineering, Kohat University of Science and Technology, Kohat 26000, Pakistan

## Abstract

Cadmium (Cd) is a widespread environmental toxic contaminant, which causes serious health-related problems. In this study, human intestinal cell line (Caco-2 cells) and normal human liver cell line (HL-7702 cells) were used to investigate the toxicity and bioavailability of Cd to both cell lines and to validate these cell lines as *in vitro* models for studying Cd accumulation and toxicity in human intestine and liver. Results showed that Cd uptake by both cell lines increased in a dose-dependent manner and its uptake by Caco-2 cells (720.15 µg mg^−1^ cell protein) was significantly higher than HL-7702 cells (229.01 µg mg^−1^ cell protein) at 10 mg L^−1^. A time- and dose-dependent effect of Cd on cytotoxicity assays (LDH release, MTT assay) was observed in both Cd-treated cell lines. The activities of antioxidant enzymes and differentiation markers (SOD, GPX, and AKP) of the HL-7702 cells were higher than those of Caco-2 cells, although both of them decreased significantly with raising Cd levels. The results from the present study indicate that Cd above a certain level inhibits cellular antioxidant activities and HL-7702 cells are more sensitive to Cd exposure than Caco-2 cells. However, Cd concentrations <0.5 mg L^−1^ pose no toxic effects on both cell lines.

## 1. Introduction

Cadmium (Cd) is one of the most concerned pollutants possessing high toxicity for both animals and plants [1]. The direct exposure of living beings, particularly human populations, to it may cause various health related problems such as Itai-Itai disease [2]. Cadmium is considered as a “guest” metal in Pb:Zn mining process and is widely dumped into the environment through various anthropogenic activities such as mining, smelting, and use of fertilizers [3]. It has been classified as a group I carcinogen by International Agency for Research in Cancer (IARC) and as a probable human carcinogen (group B1) by Environmental Protection Agency (EPA) [4]. Due to its toxic nature, it is now recognized that human exposure to Cd must either be stopped or minimized.

Cadmium salts, having greater solubility in water, can easily enter into our food chain through edible parts of the plant [5]. When grown in polluted soil, plants take up water, nutrients, and heavy metals that may then enter the food chain [6]. Khan et al. [7] found that Cd concentration in soils and food crops was above permissible limits in selected vegetables. They concluded that consuming such vegetables and food crops may result in Cd toxicity to both humans and grazing animals. Human beings are mostly exposed to Cd either by inhalation or ingestion, with oral intake being the major route. Understanding of the mechanisms of Cd absorption and circumventing the intestinal barrier is of prime interest. Available literature reports that, following oral exposure, Cd is absorbed in mammals preferentially in the duodenum and proximal jejunum [8]. After absorption Cd first reaches the liver. In liver Cd induces the production of metallothionein. After continuous hepatocyte necrosis and apoptosis, Cd-metallothionein complexes are washed into sinusoidal blood. From here, some of the absorbed Cd enters into the enterohepatic cycle via secretion into the biliary tract in the form of Cd-glutathione conjugates. Enzymatically degraded to Cd-cysteine complexes in the biliary tract, Cd again enters the small intestine [9]. It is generally assumed that, after inhalation or ingestion of typical inorganic Cd compounds and after transferring across the alveolar membranes or intestinal wall, the major fraction of Cd initially accumulates in the liver [10]. *In vitro* and *in vivo *studies have depicted that low Cd concentrations (<5.0 *μ*M) might enhance cell proliferation in a dose-dependent manner [11, 12], while its higher concentrations (>5.0 *μ*M) could induce apoptosis in many tissues and organs [13].

Apoptosis is enhanced in cells in most cases upon Cd exposure [14]. Various assays such as LDH leakage, total protein contents, and the MTT are commonly employed for the detection of cytotoxicity or cell viability upon exposure to toxic substances. The LDH leakage assay measures the lactate dehydrogenase activity in the extracellular culture medium upon the loss of intracellular LDH and is an indicator of cell membrane damage [15]. 3-[4,5-Dimethylthiazol-2-yl]-2,5-diphenyltetrazolium bromide (MTT) is a water soluble tetrazolium salt, which is converted to an insoluble purple formazan within the mitochondria. Formazan accumulates only in healthy cells and its reduction in stressed cells has been reported [16].

Like any other environmental stress, Cd stress also causes oxidative stress in cultured cells due to the production of various reactive oxygen species (ROS) such as hydrogen peroxide, superoxide radical, and hydroxyl radical. Reactive oxygen species (ROS), normally produced during the aerobic metabolism, function as second messengers involved in many cellular functions. On the other hand, when ROS level increases because of oxidant treatments and defective antioxidant systems, these highly reactive compounds and radicals become dangerous toxic agents [17]. These ROS cause imbalance between oxidants and antioxidants of the cell tissues, which leads to the numerous degenerative diseases in humans. Either to avoid or to scavenge oxidative damage, mammalian cells possess inherent antioxidant machinery, which is comprised of superoxide dismutase (SOD), catalase, and glutathione peroxidase (GPx) [18]. However, there is scarce information regarding metabolic differences in oxidatively stressed intestinal cells compared to nonstressed cells. Due to its soluble nature, Cd can readily penetrate tissues after exposure and inhibit antioxidant enzymatic system [19] by affecting the cellular thiol redox balance [20], in particular with cellular glutathione. Several studies have shown that Cd indirectly generates ROS and consequently DNA, lipid, and protein oxidation in various cell lines [21].

The Caco-2 cell line is a human intestinal cellline and HL-7702 is normal human liver cell line that have been widely used as representative models of mammalian intestinal [22] and liver cells [23]. These cells have become useful tools for the study of uptake, toxicity, and transport of nutrients [22] and heavy metals [24]. The aims of this study were to (1) determine the bioavailability of Cd in the Caco-2 cells and HL-7702 cells; (2) study the effect of Cd on the activities of antioxidant enzymes (SOD, GPx) and differentiation marker enzyme (AKP); and (3) assess the health risk of the Cd in human small intestine and liver.

## 2. Materials and Methods

### 2.1. Chemicals and Reagents

Cd(NO_3_)_2_ was purchased from Sinopharm Chemical Reagent Co., Ltd. (Shanghai, China). Dulbecco's modified Eagle medium (DMEM with glucose 4.5 g L^−1^), trypsin-EDTA, phosphate-buffered saline (PBS), fetal bovine serum (FBS), glutamine, nonessential amino acids, penicillin, and streptomycin were purchased from Gibco Life Technologies (Grand Island, NY). 3-[4,5-Dimethylthiazol-2-yl]-2,5-diphenyltetrazolium bromide (MTT) was purchased from Sigma-Aldrich (St. Louis, MO, USA). SOD, LDH, AKP, and GSH-Px testing kits were obtained from Nanjing KeyGen Biotech. Co., Ltd. (Nanjing, China). All of the other chemicals used in this study were of analytical grade purchased from local chemical suppliers. All reagents were prepared with deionized water (≤0.1 *μ*S/cm) using a Milli-Q system (Millipore, Billerica, MA). All laboratory glassware used in the experiments were soaked in 10% HNO_3_ for 24 h and subsequently rinsed with deionized water and air-dried.

### 2.2. Preparation of the Cadmium Solutions

The stock solution of cadmium nitrate was made in UHQ water and sterilized using a 0.22 mm filter. A working solution in the corresponding media (1 : 10 v/v) was prepared to give the concentrations of 0.25, 0.5, 2, 4, 8, and 10 mg L^−1^ in the culture medium.

### 2.3. Cell Culture

Caco-2 cells and HL-7702 cells were obtained from the Institute of Biochemistry and Cell Biology (SIBS, CAS, Shanghai, China) and used in assays at passage 20–33. The Caco-2 and HL-7702 cells were normally cultured in 25 cm^2^ flasks (Corning Inc., NY, USA) and maintained in high glucose (4.5 g L^−1^) DMEM, supplemented with 10% (v/v) fetal bovine serum, 1% (v/v) nonessential amino acids, 4 mM L-glutamine, and 1% (v/v) antibiotic solution (penicillin-streptomycin). The cells were maintained at 37°C in an incubator (Heraeus, BB15, Germany) with 5% CO_2_ and 95% relative humidity. After being 80% confluent, the cells were washed with phosphate-buffered saline (PBS) to remove any unattached cells. The attached cells were then harvested using a trypsin-ethylenediaminetetraacetic acid (EDTA) solution. The cells were seeded in 6-well plates at a density of approximately 10 × 10^4^ cells/mL. The medium was changed every 2 days, and the cultures were maintained for 13 days to reach the stationary growth phase and to allow for maximal functional differentiation. Confluent cultures of differentiated cells were used for further studies and were exposed to known concentrations of Cd in the culture medium and incubated for 2 and 12 h at 13 days after seeding. Cytological and biochemical assays were carried out on the cell lysate and culture medium for determination of markers of cell damage.

### 2.4. Cd Uptake

To assess the uptake of Cd(NO_3_)_2_, Caco-2 cells and HL-7702 cells, cultured in 6-well plates, were exposed to different concentrations of Cd(NO_3_)_2_ solution (0.25, 0.5, 2, 4, 8, and 10 mg L^−1^), for 2 h at 37°C. Then the cells were washed twice with ice cold PBS to remove extracellular bound Cd. The cell monolayer was lysed by the addition of 2 mL of deionized water in the well and then harvested. The lysate was put into the digestion tubes and digested with HNO_3_ (5 mL) and H_2_O_2_ (1 mL). After cooling the resultant solutions were diluted to 10 mL using deionized water. The concentrations of Cd in the final solution were determined by ICP-MS (Agilent 7500a, Agilent Technologies, CA, USA) following a standard procedure.

### 2.5. Cytotoxicity Assays

#### 2.5.1. MTT Assay

Cytotoxicity induced by Cd was assessed by MTT assay. Caco-2 cells and HL-7702 cells were seeded at a density of 5 × 10^4^ cells/well in a 96-well plate and incubated for 24 h. Cells were exposed to increasing concentrations of Cd (6 wells per concentration group plus 1 control group) for 2 h or 12 h at 37°C in an atmosphere of 5% CO_2_ in air. Control groups consist of cells in media (minus chemical), which are processed identically and incubated simultaneously as treated groups. After various time intervals (2 and 12 h), medium was removed and replaced with 50 *μ*L MTT solution (2 mg mL^−1^) for another 4 h after which medium was removed. The medium is then replaced with 100 *μ*L dimethyl lsulfoxide (DMSO), agitated for 5 min at room temperature. Finally, the absorbance was measured at 552 nm using a microplate reader (Bio-Rad-680, Bio-Rad, USA). Cell viability is expressed as a percentage of the control group. Cell MTT response (% control) was calculated from the equation:

(1) $$\begin{array}{c} \%\;\;\text{control}=\frac{{\text{Absorbance}}_{\text{treatment}}}{{\text{Absorbance}}_{\text{control}}}\times 100\%. \end{array}$$

#### 2.5.2. Lactate Dehydrogenase (LDH) Release

Cytotoxicity induced by Cd was assessed by lactate dehydrogenase (LDH) leakage into the culture medium. The activity of LDH in the medium was determined using a commercially available kit from Jiancheng Biochemical Co., Ltd. (Nanjing, China). The LDH assay is based on the conversion of lactate to pyruvate in the presence of LDH with parallel reduction of NAD to NADH. The change in the absorbance was recorded at 440 nm using a microplate spectrophotometer system (Bio-Rad-680, Bio-Rad, USA). Cell LDH release (% control) was calculated from the equation:

(2) $$\begin{array}{c} \%\;\;\text{control}=\frac{(\text{U}\;\text{LDH}/\text{mg}\;\text{cell}\;\text{protein})\;\;\text{treatment}}{\left(\text{U}\;\text{LDH}/\text{mg}\;\text{cell}\;\text{protein}\right)\;\;\text{control}}\times 100\%. \end{array}$$

### 2.6. Enzyme Assays

#### 2.6.1. Glutathione Peroxidase (GPx)

The cell glutathione peroxidase (GPx) activity was measured using the GPx cellular activity assay kit from Jiancheng Biochemical Co., Ltd. (Nanjing, China). This kit uses an indirect method, based on the oxidation of reduced glutathione (GSH) to oxidized glutathione (GSSG) catalyzed by GPx, which is then coupled with recycling GSSG back to GSH utilizing glutathione reductase and NADPH. The decrease in NADPH at 412 nm during oxidation of NADPH to NADP is indicative of GSH-Px activity. GSH-Px activity was expressed in terms of international units per mg of soluble cell proteins.

#### 2.6.2. Superoxide Dismutase (SOD)

The cell superoxide dismutase (SOD) activity was measured in Caco-2 cells and HL-7702 cells using the SOD cellular activity assay kit from Jiancheng Biochemical Co., Ltd. (Nanjing, China) based on the competition between pyrogallol oxidation by superoxide radicals and superoxide dismutation by SOD. The absorbance was measured at 550 nm using a microplate reader (Bio-Rad-680, Bio-Rad, USA). The SOD's one unit activity is defined as the amount of the enzyme required to prohibit the rate of pyrogallol autooxidation by 50%. SOD activity was expressed as international units per mg of soluble cell proteins.

#### 2.6.3. Alkaline Phosphatase

The alkaline phosphatase activity was measured by the alkaline phosphatase testing kit. This assay is based on measuring the alkaline phosphatase activity by monitoring the color change as paranitrophenol phosphate which is colorless is converted to paranitrophenol + phosphate which is yellow. Activity was determined using a microplate reader (Bio-Rad-680, Bio-Rad, USA) at 520 nm.

### 2.7. Protein Assay

Bradford [25] assay was performed in order to measure cell protein in Caco-2 cells and HL-7702 cells. Its absorbance was measured at 595 nm with a spectrophotometer (VersaMax, Molecular Devices, Sunnyvale, CA, USA).

### 2.8. Statistical Analyses

Data were analyzed using the SPSS 18.0 (SPSS Inc., Chicago, USA). Results are presented as mean ± S.D. (standard deviation). Analysis of variance (ANOVA) was performed with the least significant difference (LSD) to compare means of different Cd concentrations with control. For comparison of the different cell lines, univariate ANOVA with LSD post hoc test was used [26]. Means were considered to be significantly different if *P* values were <0.05.

## 3. Results and Discussion

In recent years, heavy metal pollution, particularly cadmium, lead, arsenic, and chromium [1], has threatened living beings, causing adverse effects on human health such as renal and testicular dysfunction and pulmonary problems. In the present study, the cytotoxic effects of Cd on a normal human liver cell line (HL-7702 cells) and human intestinal cell line (Caco-2 cells) were evaluated by studying Cd uptake by both cell lines, cytotoxicity assays, ROS scavenger enzymes, and differentiation marker enzyme (AKP).

### 3.1. Cellular Cd Uptake

**Figure 1:**
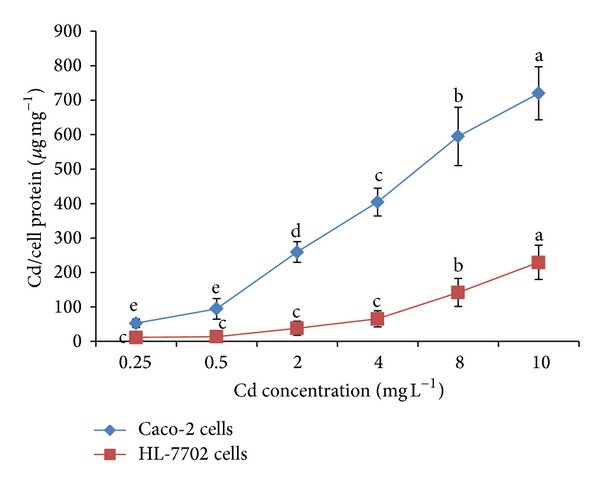
Bioavailability of cadmium (Cd) by HL-7702 and Caco-2 cells after 2 h. Values are presented as mean ± S.D.; *n* = 3. Different letters indicate significant differences at *P* < 0.05 by the least significant difference (LSD) test.

Cd accumulation in both cell lines was found significantly different due to difference in the origin of cell lines. The Cd uptake by both cell lines is shown in Figure 1. Dose-dependent rise in Cd accumulation in Caco-2 cells and HL-7702 cells could be noticed ranging from 52.3 to 720.1 and from 11.3 to 229.0 *μ*g mg^−1^ of cellular protein, respectively. Increasing Cd levels in the incubation medium were significantly (*P* < 0.05) correlated with rising cellular Cd levels. It was also observed that the uptake of the Cd at 10 mg L^−1^ by Caco-2 cells (720.15 *μ*g mg^−1^ cell protein) is higher as compared to HL-7702 cells (229.01 *μ*g mg^−1^ cell protein). Our results are in agreement with Templeton [27] who reported that Cd was absorbed from the gastrointestinal tract and was then taken up by the liver, the first organ after absorption which accumulates high Cd concentrations. Cd accumulation by the Caco-2 cells was augmented with the increasing Cd concentrations in the culture medium [28]. There can be various reasons regarding Cd uptake and its differential absorption in different organs of human body. Concentration-dependent Cd uptake in our present experiment might be due to its chemical and physical properties related to essential metals such as iron (Fe), zinc (Zn), or calcium (Ca). That is why Cd can be transported and taken up into the cells by a process referred to as “ionic and molecular mimicry” [29]. Intestinal absorption of Cd is characterized by a rapid rate of metal accumulation within the intestinal mucosa and a low rate of diffusive transfer into circulatory system [9]. In the present study we also found that the intestinal cells (Caco-2 cells) accumulated higher quantity of Cd as compared to the liver cells (HL-7702). There is a two-step process for the absorptive Cd movement from intestinal lumen into enterocytes: the first step consists of a nonspecific binding of Cd into the luminal plasma membrane and the second step consists of a transport across the luminal plasma membrane into enterocytes [30]. Much of the Cd absorbed in the intestines is delivered first to the liver through portal circulation, bound mainly to albumin, where it is taken up from the sinusoidal capillaries to the hepatocytes [31].

### 3.2. Effect of Cadmium on LDH Release and Mitochondrial Activity (MTT)

**Figure 2:**
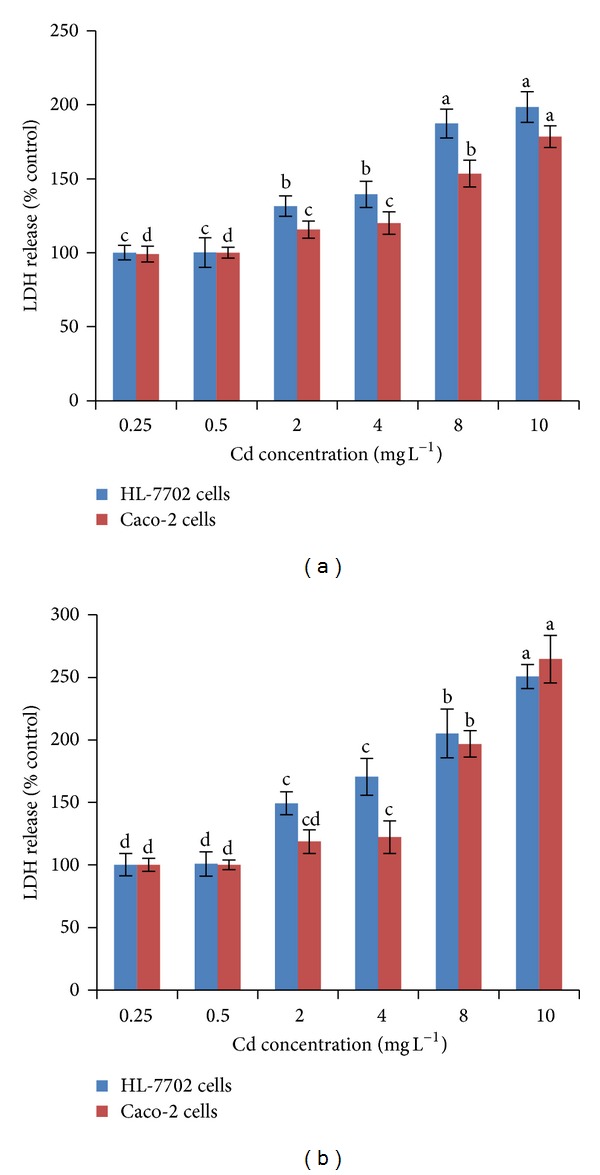
Effect of Cd on LDH release in Caco-2 and HL-7702 cells. Multiple range tests were conducted for Caco-2 and HL-7702 cells at different Cd levels after 2 h (a) and 12 h (b). Different letters indicate significant differences at *P* < 0.05 by the least significant difference (LSD) test. Values are presented as mean ± S.D.; *n* = 3.

**Figure 3:**
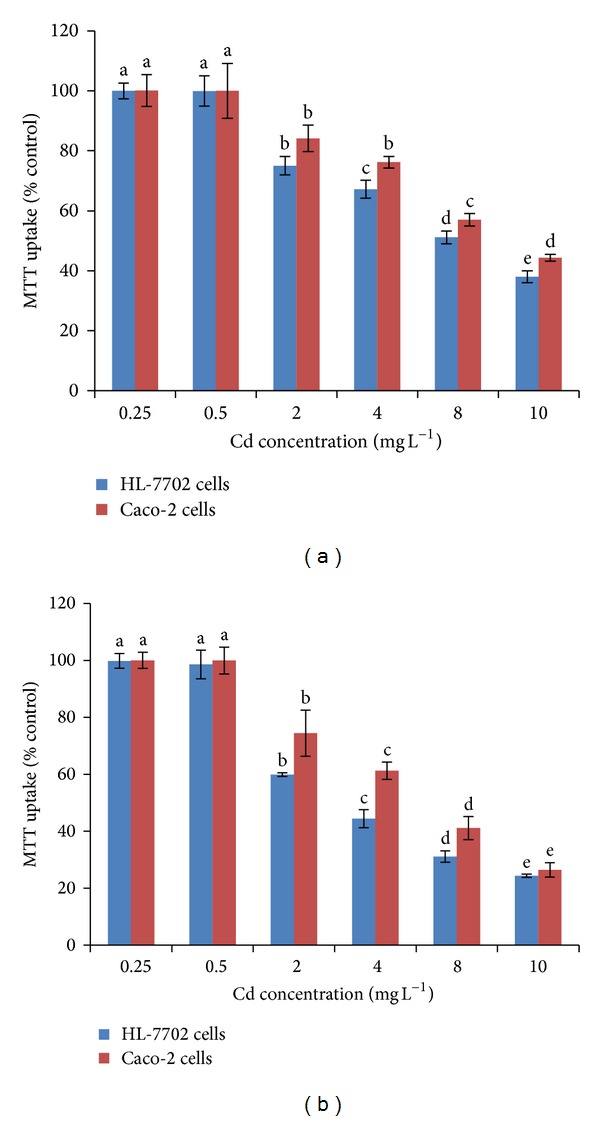
Effect of Cd on MTT assay in Caco-2 and HL-7702 cells. Multiple range tests were conducted for Caco-2 and HL-7702 cells at different Cd levels after 2 h (a) and 12 h (b). Different letters indicate significant differences at *P* < 0.05 by the least significant difference (LSD) test. Values are presented as mean ± S.D.; *n* = 3.

The results obtained from the cytotoxicity assays indicate that there are differences between the two cell lines concerning their sensitivity to Cd exposure. HL-7702 cells appear to be more sensitive as indicated by the LDH and MTT assays (Figures 2 and 3). LDH release assay is an important method to assess cell membrane stability [13]. As shown in Figures 2(a) and 2(b), there was an increase in LDH release when Caco-2 cells and HL-7702 cells were exposed to different concentrations for 2 and 12 h. Increased concentrations and time of Cd exposure resulted in a significant increase of LDH release in culture media. At Cd concentrations greater than 0.5 mg L^−1^, a significant (*P* < 0.05) increase in LDH release was observed for both cell lines, as compared with their respective controls. It was found that Cd dosage increased the LDH release from 99% to 178.5% in Caco-2 cells and from 100% to 198.4% in HL-7702 cells at the incubation time of 2 h. After 12 h, this increase was much larger at 10 mg L^−1^ both in Caco-2 cells (264.6%) and HL-7702 cells (250.7%). In terms of LDH release, for both cell lines, Cd levels and exposure time significantly correlate (*P* < 0.05) with each other. The large increase in LDH release might also be due to cell rounding and loss of adherence found at the concentration of 10 mg L^−1^ Cd. This noticeable increase of LDH release might be due to the increase of necrotic cell death as scavenging enzymes could be depleted by relatively high concentrations of Cd. Morphological changes (loss of adherence and cell rounding) based on 10 mg L^−1^ Cd might be associated with loss of intercellular contacts and break down of desmosomes [26].

The MTT assay is mainly based on the enzymatic conversion of MTT in the mitochondria. It has been suggested that Cd disrupts mitochondrial function both *in vivo* [32] and *in vitro* [33]. Apoptosis mediated by mitochondria may be relevant in metal-induced cell death. Mitochondrial activity was significantly decreased by increasing concentrations and time of Cd exposure to both cells (Figures 3(a), and 3(b)). MTT assay was very low (<3%) in both cell lines treated with 0–0.5 mg L^−1^ Cd. A significant decrease in MTT assay, compared to control cells, was noted in Caco-2 cells and HL-7702 cells subjected to Cd for 2 and 12 h. Initial cytotoxicity was observed for both cell lines at 2 mg L^−1^ Cd. Cadmium dosage decreased cell viability by 84% and 44% in Caco-2 cells and by 75% and 38% in HL-7702 cells after 2 h while the percentage further decreased after 12 h in both Caco-2 cells (74% and 26%) and HL-7702 cells (60% and 24%) under different concentrations of Cd (2 mg L^−1^ and 10 mg L^−1^), as compared to their respective controls. These results indicated that Cd was able to induce cytotoxicity in a concentration- and time-dependent manner in the Caco-2 cells and HL-7702 cells. Similar findings were also reported by Nemmiche et al. [34] and Fotakis and Timbrell [15]. Cadmium toxicity primarily resulted from the binding of Cd to thiol groups in mitochondria, causing mitochondrial dysfunction and subsequent injury. Furthermore, Cd may enter mitochondria through Ca uniporter and interact with thiol groups of adenine nucleotide translocator (ANT) to induce the Cytochrome c release and apoptosis. Xie et al. [23] also reported that Cd-induced apoptosis was dependent on Cd dose and exposure time in both normal (HL-7702) and tumor cells (Raji cells). Furthermore they [23] observed that Cd-induced Ca elevation was attenuated in HL-7702 cells coincubated with a Ca chelator. Therefore, Cd-induced apoptosis was mediated by the release of Ca from intracellular Ca storage but not an influx of extracellular Ca.

### 3.3. Effect of Cd on Antioxidant Enzymes Activity

Cadmium promotes an early oxidative stress and thus contributes to the development of serious pathological conditions because of its long retention in some tissues [35]. Physiologically, SOD is an important defense enzyme, which converts O_2_ to H_2_O_2_ and thus protects against superoxide-induced damage [36]. GSH-Px, in particular, is highly dependent on glutathione concentration. The antioxidant enzymes (SOD, GPx) activities in Caco-2 cells and HL-7702 cells exposed to different concentrations of Cd for 2 h are shown in Table 1. Significant changes in antioxidant enzymes activity were observed in both cell lines with the increase in Cd level, as compared with controls. Cd had no significant influence (*P* > 0.05) on SOD activity in both cell lines when the concentration was below 0.5 mg L^−1^. However, the SOD activity was significantly decreased (*P* < 0.05) when the concentration of Cd was more than 0.5 mg L^−1^. SOD activity of Caco-2 cells and the HL-7702 cells was decreased by 61.1% and 42.7%, respectively at 10 mg L^−1^ Cd level, as compared to their respective controls. The results of GPx activity showed a similar trend to SOD activity. The results showed that GPx activity in Cd-treated Caco-2 cells and HL-7702 cells ranged from 96% to 15.3% and 97.8% to 19.7%, respectively at 0.25 mg L^−1^ and 10 mg L^−1^ from control. Our results showed that antioxidant enzyme activities influenced by Cd in both cell lines differed significantly from their controls. In response to Cd stress, the activities of SOD and GPx in HL-7702 cells were considerably higher than those of Caco-2 cells.

According to our findings, the major toxic effects of increasing doses of Cd concentration involve decreased antioxidant enzyme levels. We presume that Cd also enters the mitochondria and inhibits the activities of many enzymes by binding to their −SH groups or by inhibiting the protein synthesis [37]. Cadmium binding to cysteine in reduced glutathione (GSH) results in the inactivation of GSH-Px, which, therefore, fails to convert H_2_O_2_ to water [38, 39]. Moreover, there is an increasing evidence that Cd interacts with Se and disrupts GSH-Px activity. This probably is the consequence of the intracellular accumulation of ROS with subsequent development of tissue injury.

### 3.4. Effect of Cd on Differentiation Marker Enzyme (AKP)

Cadmium exposure had a significant (*P* < 0.05) effect on AKP activity in Caco-2 cells and HL-7702 cells (Table 1). AKP activity was highest in Caco-2 cells (97.6%) and HL-7702 cells (95.5%) when treated with 0.25 mg L^−1^ Cd, while at 10 mg L^−1^ its activity was decreased by 66.1% and 27.01%, respectively.

Our results showed that differentiation marker enzyme (AKP) was significantly decreased in both cell lines after 2 h Cd exposure. The inhibition of alkaline phosphatase activity has been already reported by others [40–42]. Also, El-Demerdash et al. [43] revealed that Cd *in vitro* caused significant inhibition of AKP activity in human plasma. The decrease in AKP activity may be due to changes in the permeability of plasma membrane in addition to changes in the balance between synthesis and degradation of enzyme [40]. Also, Lakshmi et al. [44] reported that the inhibition of AKP may be due to the breakdown of the membrane transport system and an inhibitory effect on cell growth and proliferation.

## 4. Conclusions

This study concludes that Caco-2 and HL-7702 cell models are in good agreement with the *in vivo *experiment after oral ingestion of Cd: low rate being transferred from the lumen into the body, higher accumulation in the intestinal cells than the liver cells. These results demonstrated that Cd is able to induce oxidative damage and apoptosis in both cell lines when its concentration exceeds 0.5 mg L^−1^. Finally, our findings suggest that HL-7702 cells are more sensitive to Cd exposure than Caco-2 cells at the same concentration and exposure duration.

## Figures and Tables

**Table 1:** Activities of antioxidants enzymes (SOD, GPX) and differentiation marker enzyme (AKP) in Caco-2 cells and HL-7702 cells at different Cd levels (after 2 hr exposure).

Parameters	Cell lines	Cd concentration (mg L^−1^)						
		0	0.25	0.5	2	4	8	10
GPx (U mg^−1^ cell protein)	Caco-2 cells	198.7 ± 0.7^a^	190.9 ± 2.6^b^	183.9 ± 3.2^c^	133.7 ± 0.8^d^	70.1 ± 1.1^e^	48.09 ± 0.9^f^	30.3 ± 0.7^g^
	HL-7702 cells	306.9 ± 2.3^a^	300.2 ± 8.7^ab^	295.6 ± 5.3^b^	207.2 ± 1.1^c^	144.9 ± 0.8^d^	100.3 ± 0.6^e^	60.4 ± 0.6^f^
SOD (U mg^−1^ cell protein)	Caco-2 cells	26.2 ± 1.8^a^	25.1 ± 3^a^	22.91 ± 2.4^ab^	21.8 ± 0.5^bc^	21.7 ± 0.9^bc^	19.0 ± 2^cd^	15.9 ± 0.3^d^
	HL-7702 cells	26.0 ± 1.8^a^	24.5 ± 0.6^a^	24.0 ± 0.2^a^	21.4 ± 1.0^b^	20.03 ± 2.0^b^	15.2 ± 0.5^c^	11.1 ± 1.1^d^
AKP (%Ck)	Caco-2 cells	100 ± 0.1^a^	97.6 ± 3.3^b^	95.3 ± 3.6^b^	83.1 ± 0.7^c^	79.41 ± 0.7^d^	75.0 ± 1.0^e^	66.1 ± 0.6^f^
	HL-7702 cells	100 ± 0.4^a^	95.5 ± 5.1^b^	90.7 ± 3.7^c^	65.32 ± 0.9^d^	45.97 ± 1.0^e^	28.31 ± 1.3^f^	27.01 ± 1.0^f^

Multiple comparison tests were for Caco-2 cells and HL-7702 cells at different Cd levels and different letters indicate significant differences at *P* < 0.05 as calculated by the least significant difference (LSD) test. Values are presented as mean ± S.D.; *n* = 3.
